# Independent Audit of Asylum Accounts

**Published:** 1917-02-10

**Authors:** 


### Independent Audit of Asylum Accounts.

The Court of Appeal in Ireland have decided a most important point in Public Health administration. In the case of *Rex* (*Local Government Board*) v. *McLaughlin* an order was sought against the respondent requiring him to attend upon and assist the auditor in checking the accounts of an asylum committee to which he was a clerk. It seems that a practice has grown up of Local Government officials assisting auditors in vouching and checking accounts and payments, and the Local Government Board has supported the auditors in making a demand for this service on the part of local officers. On this particular respondent refusing, the Local Government Board made an order in which the duty was imposed on him in the most specific terms; but this, too, McLaughlin refused to recognise. Now both in the King's Bench Division and in the Court of Appeal the Board's procedure has been held bad; and it has been laid down that an audit of the accounts of a public body should be carried out by an independent auditor, and that there is no warrant in law for the giving of any assistance to him by a local official or for his accepting any such assistance. It is his duty to audit, to examine, and to exercise his mind and judgment upon every book, account, and document, and he cannot do this if these are held by a second person. The Court also have agreed that the accounting officer in the case of a lunatic asylum is the resident medical superintendent, not the clerk.

